# Immune Landscape Variation in Antineutrophil Cytoplasmic Antibody-Associated Vasculitis Circulation Before and After Plasmapheresis by Single-Cell Transcriptome

**DOI:** 10.1155/mi/5531382

**Published:** 2025-04-10

**Authors:** Youzhou Tang, Qingtai Cao, Jishi Liu, Quan Zhuang

**Affiliations:** ^1^Department of Nephropathy and Rheumatology, The 3rd Xiangya Hospital, Central South University, Changsha, Hunan, China; ^2^The Critical Kidney Disease Research Center, Central South University, Changsha, Hunan, China; ^3^Transplantation Center, The 3rd Xiangya Hospital, Central South University, Changsha, Hunan, China; ^4^Research Center of National Health Ministry on Transplantation Medicine, Changsha, Hunan, China

**Keywords:** antineutrophil cytoplasmic antibody-associated vasculitis (AAV), flow cytometry, monocyte, plasmapheresis, single-cell RNA sequencing

## Abstract

Antineutrophil cytoplasmic antibody (ANCA)-associated vasculitis (AAV) is a group of autoimmune diseases characterized by inflammation and destruction of small blood vessels. AAV could be fatal if left untreated. Prompt diagnosis and treatment are crucial to protect AAV-related organs and tissue. Plasmapheresis, a therapeutic intervention aimed at removing harmful substances from the blood, devotes benefits to AAV treatment. However, the specific immune mechanism underlying its effectiveness remains unclear. In our research, we used single-cell RNA sequencing (scRNA-seq) to study the variation of peripheral blood mononuclear cells (PBMCs) before and after plasmapheresis in AAV patients. From this work, we explored a novel method for monocyte classification. In addition, flow cytometry was used to detect the relationship between the monocyte clusters and AAV activity under the new monocyte clustering method. Our scRNA-seq results revealed significant changes in monocyte clusters following treatment, which could be classified into three clusters (CD14+ monocytes, FCGR1A+ monocytes, and FCGR3A+ monocytes). In addition, our flow cytometry results showed that FCGR3A+ (CD16+) monocytes were positively correlated with AAV activity, whereas FCGR1A+ (CD16-CD64+) monocytes were negatively correlated with AAV activity. This may be related to the different biological effects of CD16 and CD64 on monocytes after interacting with the Fc region of ANCAs. In conclusion, our research sheds light on the immune landscape of AAV before and after plasmapheresis, identifying specific monocyte clusters linked to disease activity. These findings offer insights for novel monitoring methods and therapeutic targets in AAV.

## 1. Introduction

Antineutrophil cytoplasmic autoantibody (ANCA)-associated vasculitis (AAV) includes granulomatosis with polyangiitis (GPA), microscopic polyangiitis (MPA), and eosinophilic granulomatosis with polyangiitis (EGPA) [1, 2]. These small vessel vasculitis are complex immune-mediated diseases resulting fjrom the interplay of highly specific immune responses. This response targets the normally cryptic epitopes of neutrophil granule proteins, producing a large number of autoantibodies called ANCA. ANCAs are serum markers of AAV, which can be divided into cytoplasmic antineutrophil cytoplasmic autoantibody (cANCA) and perinuclear antineutrophil cytoplasmic autoantibody (pANCA) forms under immunofluorescence. Myeloperoxidase (MPO) and proteinase 3 (PR3) are the main target antigens of ANCA since ANCAs can be divided into MPO-ANCA and PR3-ANCA by ELISA [3]. ANCA formation is one of the hallmarks of AAV. ANCA targets antigens found primarily in neutrophils and monocytes, and these autoantibodies cause tissue damage by interacting with neutrophils, monocytes, and endothelial cells sensitized by preexcitation. AAV can damage a variety of organs, especially the lung and kidney [3]. If there is no timely and effective treatment to the acute inflammation of AAV, the mortality rate will be extremely high. The management of AAV is challenging due to its recrudesce and potential for severe complications [3]. This is specifically reflected in the assessment of disease activity and risk of recurrence, and the need for more effective and safer targeted therapies.

Currently, the treatment of AAV includes immunosuppressive therapy and plasmapheresis [4]. Plasmapheresis offers the advantage of rapidly reducing pathogenic ANCAs, immune complexes, and inflammatory cytokines in circulation, potentially ameliorating the acute phase of the disease [5, 6]. Despite its clinical utility, the precise cellular and molecular mechanisms underlying plasmapheresis' therapeutic effects in AAV remain poorly defined. The in-depth study of its mechanism can help provide insights into novel monitoring and therapeutic targets for AAV. Therefore, the aim of this study was to elucidate the changes in peripheral blood mononuclear cells (PBMCs) before and after plasmapheresis in AAV patients and find the key cell clusters.

The single-cell RNA sequencing (scRNA-seq) is a technique for sequencing and analyzing the transcriptome at the level of a single cell. Traditional RNA sequencing, which is performed on a multicell basis, actually obtains the mean of the transcriptome information in a bunch of cells. scRNA-seq technology can detect the heterogeneity information that cannot be obtained by traditional RNA sequencing, thus solving this problem. We used scRNA-seq technology to analyze the heterogeneity of PBMCs before and after plasmapheresis, providing a high-resolution view of immune cell subsets involved in AAV. Our analysis revealed significant changes in monocyte populations post-treatment, leading to a novel classification into three distinct clusters: FCGR1A+ (CD16-CD64+) monocytes, FCGR3A+ (CD16+) monocytes, and CD14+ (CD16-CD64-) monocytes. In addition, the correlation between these monocyte subsets and AAV activity was analyzed by flow cytometry. The proportions of CD16+ monocytes positively correlated with disease activity, while CD16-CD64+ monocytes exhibited a negative correlation. These associations highlight the potential of monocyte profiling as a biomarker for AAV activity and treatment response. These results provide new prospects for improving the assessment methods of disease activity and recurrence risk, as well as for the development of new safer and more effective targeted therapeutic strategies.

## 2. Materials and Methods

### 2.1. Sample Collection and Processing

A total of four peripheral blood samples were collected from two AAV patients before and after plasmapheresis in the Department of Nephrology, Third Xiangya Hospital, Central South University for scRNA-seq analysis. Fresh peripheral blood samples were rapidly transported to the Singleron laboratory. PBMCs were isolated using density gradient centrifugation. Red blood cells were removed by adding 2 mL of GEXSCOPE red blood cell lysis buffer (RCLB, Singleron) for 10 min. The solution was centrifuged at 500 × *g* for 5 min, followed by suspension in phosphate-buffered saline (PBS). Blood samples were then centrifuged at 400 *g* for 5 min at a temperature of 4°C, and the resulting supernatant was discarded. PBMCs were isolated through centrifugation at 400 *g* for 10 min at a temperature of 4°C after removing red blood cells. The supernatant was discarded, and the PBMCs were resuspended using phosphate buffered saline to achieve a single-cell suspension. Finally, Trypan Blue staining was performed on the samples, and cell viability was assessed under microscopic examination. In addition, after the analysis of scRNA-seq data, peripheral blood samples were collected from ten AAV patients in the Department of Nephrology, Third Xiangya Hospital, Central South University for flow cytometry analysis to explore the correlation between cell populations of interest and AAV activity. Patients were excluded if they were younger than 18 years of age or if they had other conditions that significantly affected the study results. All sample acquisition procedures were conducted in accordance with relevant medical ethical standards. According to the “Standard of Diagnosis and Treatment of Rheumatic Diseases (2021)” prepared by the Chinese Rheumatology Association, ANCA is the most common autoantibodies in the serum of AAV patients, and it is an important basis for the diagnosis of AAV. The main pathological changes of AAV are inflammation and necrosis of the small vessel wall. Fibrinoid necrosis of the vessel wall is a characteristic pathological change of vasculitis, which is the gold standard for the diagnosis of AAV. Plasmapheresis is indicated in patients with either new or recurrent AAV and in those with a serum creatinine level of more than 500 μmol/L due to rapidly progressive glomerulonephritis or in those requiring dialysis. Severe alveolar hemorrhage is also an indication for plasma exchange. Patients also received glucocorticoids and cyclophosphamide during plasmapheresis.

### 2.2. scRNA-seq Process

A solution of single cells (2 × 10^5^ cells/mL) in PBS was loaded into a microwell chip using the Singleron Matrix Single Cell Processing System. Barcoding Beads were then retrieved from the chip, and mRNA captured by these beads underwent reverse transcription to generate cDNA, which was subsequently amplified via PCR. The amplified cDNA fragments were fragmented and ligated with sequencing adapters before being used to construct scRNA-seq libraries following the GEXSCOPE Single Cell RNA Library Kits protocol (Singleron) [7]. These individual libraries were diluted to 4 nM, pooled together, and sequenced on an Illumina novaseq 6000 platform with paired end reads of 150 bp length.

### 2.3. Processing of Raw Read Data

The gene expression matrices were generated from scRNA-seq raw reads using the CeleScope pipeline (https://github.com/singleron-RD/CeleScope). Initially, low-quality reads were eliminated by applying Cutadap in conjunction with CeleScope to trim poly-A tail and adapter sequences. Subsequently, cell barcode and UMI information were extracted. The STAR [8] tool was used for mapping the reads to the GRCh38 reference genome (ensembl version 92 annotation). To obtain UMI counts and gene counts per cell, featureCounts [9] software was utilized. These counts were then used to create expression matrix files for subsequent analysis.

### 2.4. Preprocessing of scRNA-seq Data

In the “Seurat” R Package, low quality and adhesive cells were excluded by removing any cells expressing less than 200 genes, more than 4000 genes, and more than 15% mitochondrial gene expression. The gene expression matrix was transformed using the function “NormalizeData” for better interpretability. The “IntegrateData” function was used for data merging and batch effects removal. After that, principal component analysis (PCA) and UMAP dimensionality reduction clustering was performed.

### 2.5. Differentially Expressed Gene Analysis

To identify differentially expressed gene (DEG), we employed a nonparametric approach based on the Wilcoxon rank-sum test using the “Seurat” R package's “FindMarkers/FindAllMarkers” function. DEGs were defined as those with an average log (Fold Change) greater than 0.25. For cell type annotation within each cluster, we integrated the expression of classical markers found among the DEGs with established literature. The expression of markers for each cell type was visualized using heatmaps, dot plots, and violin plots generated by “Seurat” R Package. Manual curation was performed to filter out doublets and low-quality cells identified as expressing markers of different cell types.

### 2.6. Cell Type Annotation

Classical biomarkers of PBMCs were used to annotate cell clusters after clustering: T cells (CD3D, CD3E, CD3G, CD8A, CD8B, CCR7, LEF1, GZMH, GZMK), B cells (CD79A, CD79B, MS4A1, TCL1A, CD22, CD19), monocytes (CD14, S100A8, S100A9, FCGR3A, MS4A7), NK cells (NKG7, GZMB, GZMA, CST7), DC (FCER1A, CST3, CLEC10A), and platelets (PPBP, GP9, ITGA2B, PF4).

### 2.7. Gene Ontology/Kyoto Encyclopedia of Genes and Genomes Enrichment Analysis

To explore the corresponding functions of the DEGs, we conducted gene ontology (GO) and Kyoto Encyclopedia of Genes and Genomes (KEGG) analyses using the “clusterProfiler” R package. GO and KEGG enrichment analyses are two bioinformatics methods used to understand the function and biological significance of a collection of genes or proteins. GO is a standardized vocabulary for describing the functions of genes and their products, which includes three main ontologies: molecular function (MF), biological process (BP), and cellular component (CC). KEGG is a database for understanding biological systems and their interactions, mainly including signal transduction pathways. GO terms and KEGG pathways with an adjusted *p*-value less than 0.05 were considered significantly enriched.

### 2.8. Pseudotime Trajectory Analysis and Cell–Cell Interaction Analysis

We reconstructed cell differentiation trajectories using “Monocle2” R package, ordering cells by their temporal and spatial differentiation sequence based on DEGs. Dimensionality reduction was performed using the “reduceDimension” function within the DDRTree algorithm. Pseudotemporal trajectory analysis was visualized using the plot_cell_trajectory function. Cell–cell interactions were analyzed using the “CellChat” R package. “CellChat” is an R package for analyzing cell-to-cell communication. It can identify and visualize communication networks between cells, helping to understand how cells interact through signaling.

### 2.9. Flow Cytometry Analysis

Flow cytometry was performed to quantify monocyte subsets in PBMC samples. Cells were stained with fluorescently labeled antibodies against CD14, CD16, and CD64. Data acquisition was done using a flow cytometer (BDcanto II), and at least 50,000 events were recorded per sample. Data analysis was carried out using FlowJo software, with gating strategies to identify monocyte populations as defined by the scRNA-seq results. Pearson correlation coefficient was calculated to assess the relationship between monocyte subsets and disease activity, measured by the Birmingham Vasculitis Activity Score (BVAS) [10].

### 2.10. Statistical Analysis

Statistical analyses were performed using R software. *p*-value  < 0.05 were considered statistically significant.

## 3. Results

### 3.1. Preprocessing and Quality Control of scRNA-Seq Data

**Figure 1:**
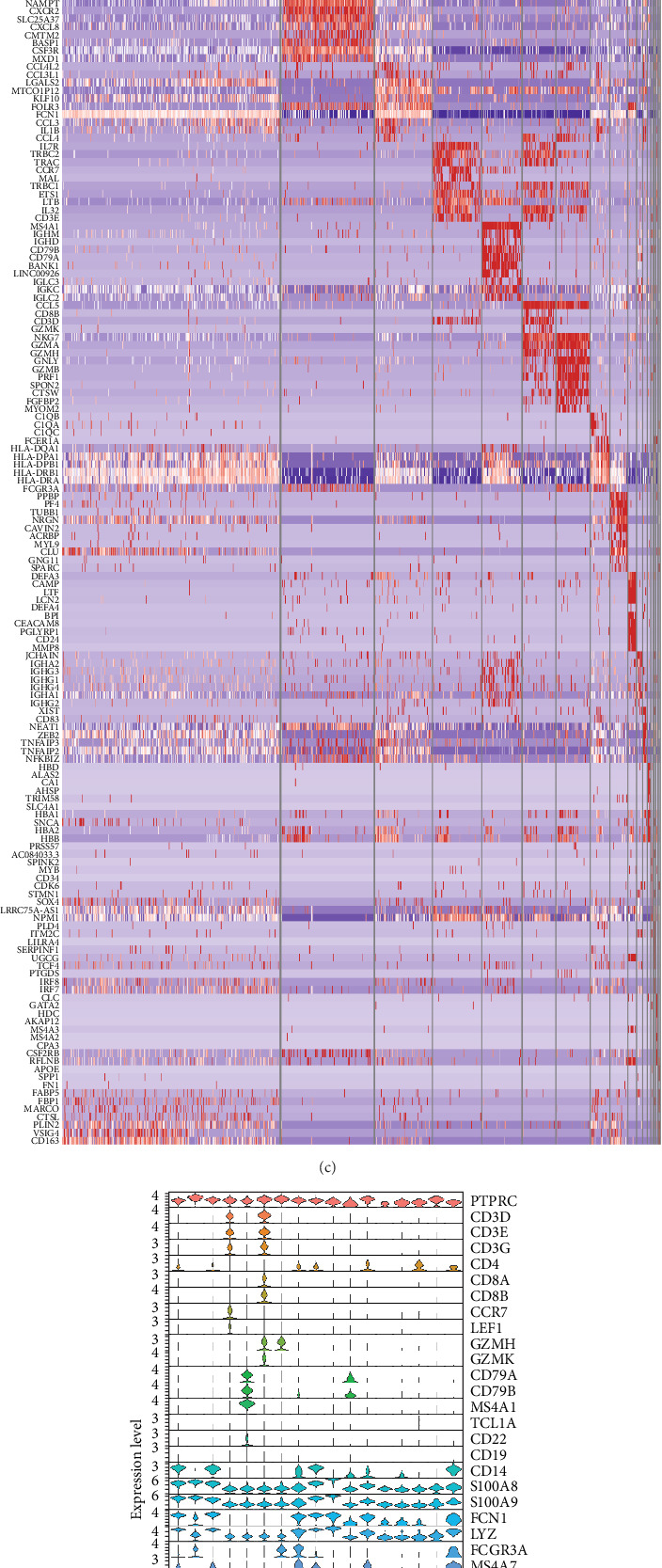
Presentation of single-cell RNA sequencing (scRNA-seq) data of peripheral blood mononuclear cells (PBMCs). (A) UMAP plot of PBMCs. (B) UMAP plots of PBMCs before and after plasmapheresis. (C) Heatmap of differentially expressed genes between clusters. (D) Violin plot of PBMC biomarkers in clusters. (E) UMAP plot of PBMC biomarker.

After quality control measures were applied using the “Seurat” R Package, a total of 28,861 cells met our criteria for further analysis (Supporting Information Figure S1). PBMCs were clustered into 17 clusters using PCA and UMAP by “integrated_snn_res = 0.3” (Figure 1A, Supporting Information Figure S2). The choice of this resolution was based on a balance of granularity and biological interpretability. Clustering was already stable at this resolution. In addition, we visualized the PBMCs landscape of both groups by UMAP plots (Figure 1B).

### 3.2. Immunological Landscape of PBMCs in AAV Before and After Plasmapheresis

**Figure 2:**
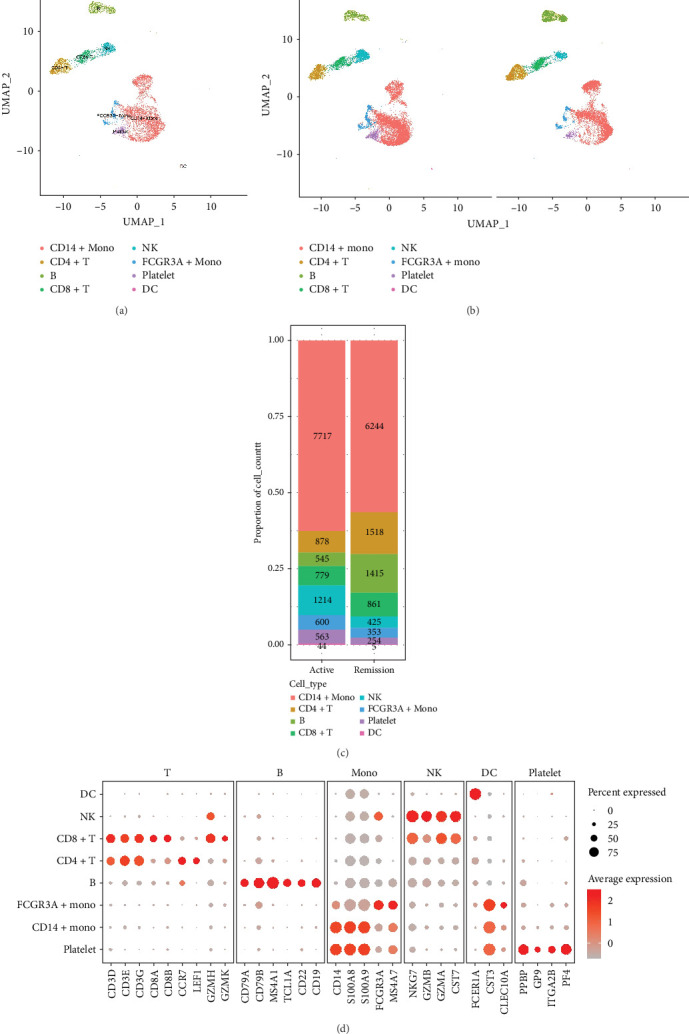
Annotation results of peripheral blood mononuclear cells (PBMCs). (A) Cell-type annotation of PBMCs. (B, C) Changes in PBMCs in the active phase before treatment and in the remission phase after treatment. (D) Biomarkers of PBMC are shown in violin plots after annotation.

We identified DEGs across different cell populations (Figure 1C). The expression profiles of classical markers, which were consistent with the literature, were used to annotate cell types within each cluster (Figure 1D,E). Visualization of these markers was achieved through the generation of heatmaps, dot plots, and violin plots. We also manually curated the dataset to exclude doublets and low-quality cells that were expressing markers characteristic of multiple cell types (Figure 2A). The results showed that there was a reduction in CD14+ monocytes, natural killer (NK) cells, FCGR3A+ monocytes, platelets, and dendritic cells (DCs) after plasma exchange. On the other hand, the proportion of CD4+T cells and B cells was increased (Figure 2B,C2 Classical biomarkers of PBMCs are shown in Figure 2D. At the same time, we also showed the variation of each sample (Supporting Information Figure S3A).

### 3.3. Annotation and Pseudotime Trajectory of Monocytes

**Figure 3:**
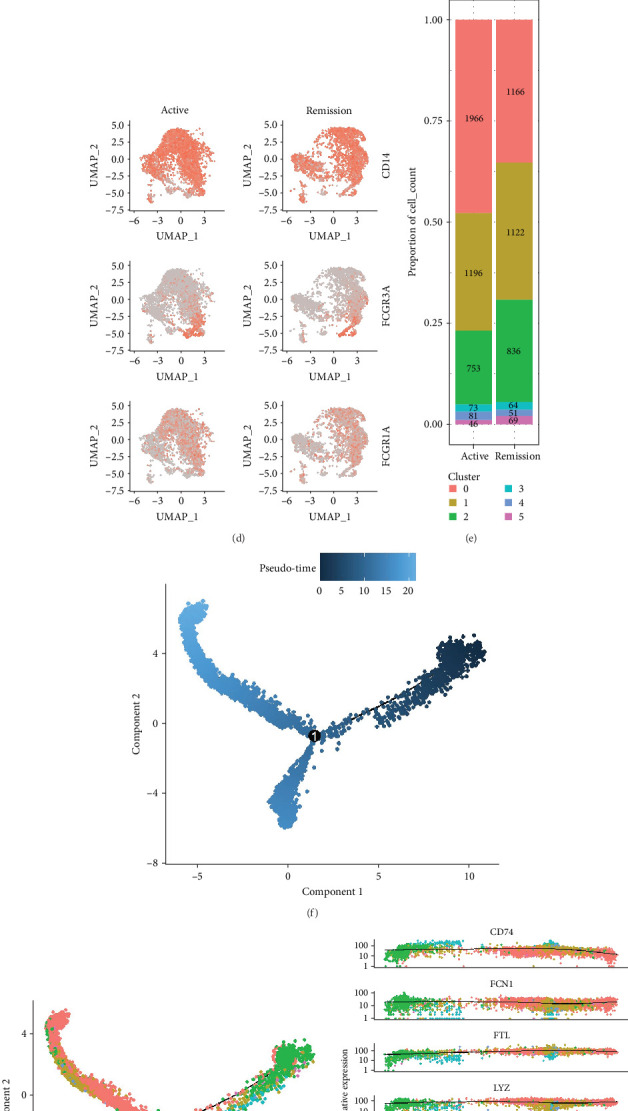
Clustering and pseudo-temporal analysis of monocytes. (A, B) The clustering of monocytes and the changes before and after treatment. (C, D) Specific biomarkers for monocyte clustering. (E) Changes in the proportion and number of monocyte clusters before and after treatment. (F–I) Pseudo-time-series analysis of monocytes.

**Figure 4:**
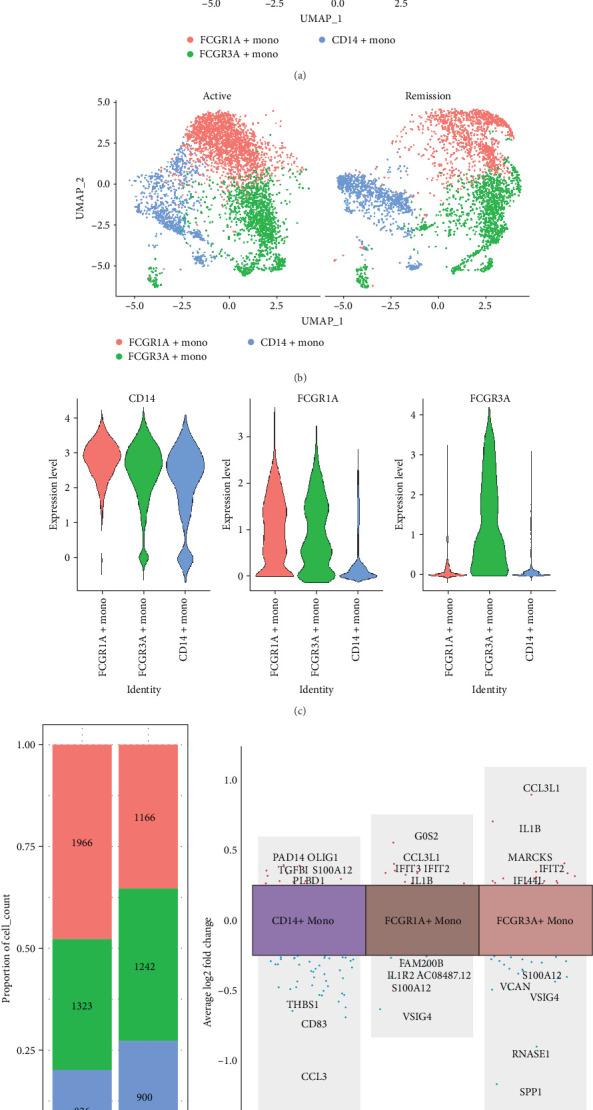
Annotation of monocyte clusters. (A, B) Annotated monocytes and their changes before and after treatment. (C) Biomarkers of monocyte clusters. (D) Changes in the proportion and number of monocyte clusters. (E) Differentially expressed genes in monocytes before and after treatment.

Monocytes could be divided into six clusters (Figure 3A,B, Supporting Information Figure S3B). The proportions and cell counts of these six clusters were shown (Figure 3E). Three significant markers, CD14, FCGR3A, and FCGR1A, were used to define new monocyte clusters (Figures 3C,D, 4A–C). These biomarkers distinguished the first three most dominant monocyte subgroups, considering the very small number of the latter subgroups. FCGR1A+ monocytes and FCGR3A+ monocytes decreased after plasmapheresis, and CD14+ monocytes increased after plasmapheresis (Figure 4D). The application of Monocle2 allowed us to reconstruct the cell differentiation trajectories, revealing a developmental continuum among the analyzed cells (Figure 3). The state of the cell and its progression during pseudotime were elucidated. Differential genes of three monocyte subsets before and after treatment were explored by differential analysis (Figure 4E). The top five upregulated and downregulated DEGs for each cell subset are shown. The results showed that monocytes had a significant decrease in the expression of inflammation-related genes after plasmapheresis.

### 3.4. GO/KEGG Enrichment Analysis

**Figure 5:**
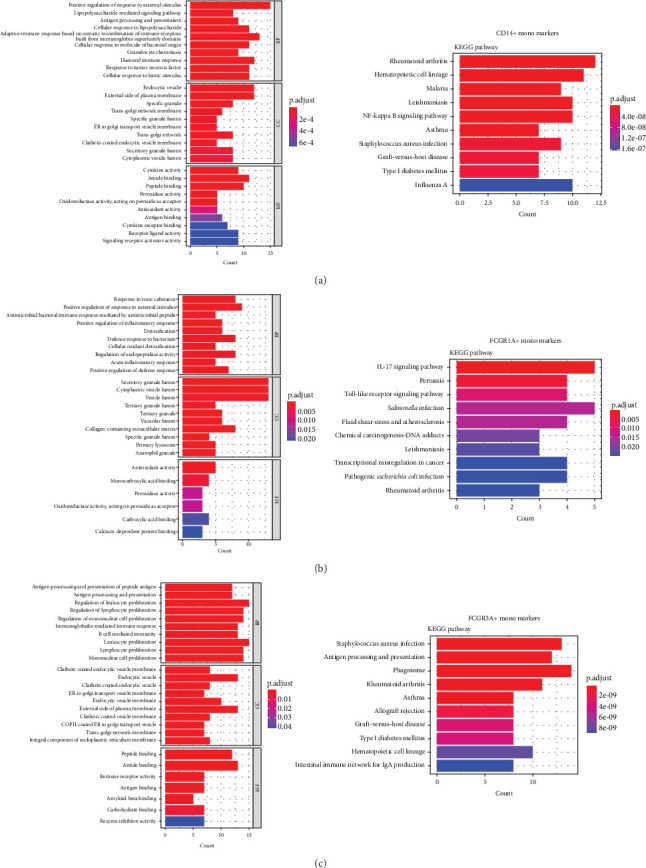
Gene Ontology (GO)/Kyoto Encyclopedia of Genes and Genomes (KEGG) enrichment analysis of biomarkers for monocyte clusters. (A) enrichment analysis of CD14+ monocyte markers; (B) enrichment analysis of FCGR1A+ monocyte markers; and (C) enrichment analysis of FCGR3A+ monocyte markers.

Enrichment analysis was performed for the three monocyte clusters. Significant pathways were uncovered through GO and KEGG analyses (Figure 5). We identified several enriched GO terms within the categories of MF, BP, and CC, which suggested a role for the DEGs. KEGG pathway analysis further highlighted the involvement of these DEGs in critical biological pathway, shedding light on their potential functional implications. In addition, we also performed enrichment analysis of DEGs before and after plasmapheresis (Supporting Information Figures S4 and S5).

### 3.5. Cell–Cell Interaction Analysis

**Figure 6:**
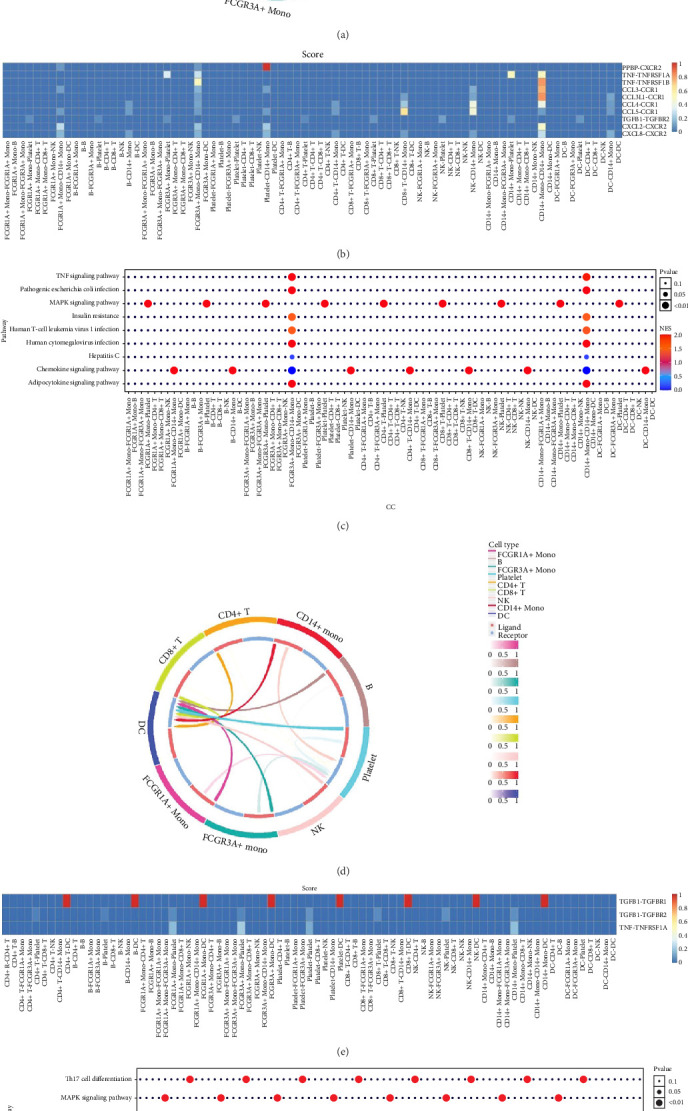
Cell–cell interaction analysis. (A–C) Cell–cell interaction analysis before treatment. (D–F) Post-treatment cell–cell interaction analysis.

Furthermore, by employing CellChat, we mapped a network of cell–cell interactions, identifying key signaling pathways that mediate communication between cell types (Figure 6). The reannotation of PBMCs is described in Supporting Information Figure S6, based on the new classification of monocytes. (Supporting Information Figure S6) These results are presented by cell interaction maps and heat maps. The results showed that PMBCs mainly interacted with CD14+ monocytes before plasmapheresis. After plasmapheresis, PBMCs mainly interact with DCs.

### 3.6. Correlation Between Monocyte Subsets and Antineutrophil Cytoplasmic Antibody-Associated Vasculitis Activity

**Figure 7:**
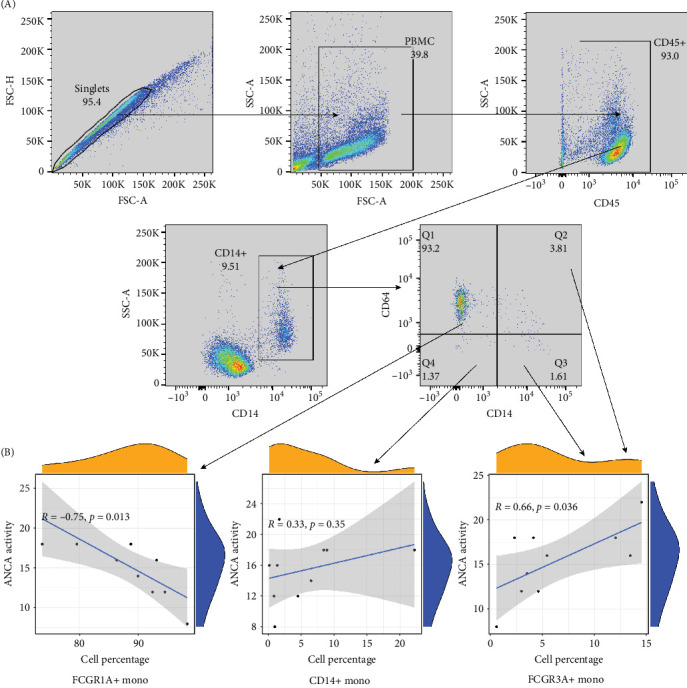
Correlation between monocyte clusters and AAV activity. (A) The gate setting strategy of flow cytometry. (B) Correlation between monocyte clusters and AAV activity.

Flow cytometry analysis was conducted on PBMC samples, and monocyte subsets were quantified using antibodies against CD14, CD16, and CD64 (Figure 7A). The frequencies of these subsets were visualized in scatter diagrams, demonstrating a correlation with the monocyte clusters (Figure 7B). Result suggested that CD16+ monocytes (FCGR3A+ monocytes) positively correlate with AAV activity (*R* = 0.66, *p* =0.036), while CD16-CD64+ monocytes (FCGR1A+ monocytes) negatively correlate (R = −0.75, *p* =0.013). We presented more clinical scoring details of the BVAS (Supporting Information Table S1). Demographic and other disease information was also provided (Supporting Information Table S2).

## 4. Discussion

In this study, we characterized the changes in the immune landscape of PBMCs in AAV patients before and after plasmapheresis by scRNA-seq. We were concerned that the proportion of both CD14+ monocytes and FCGR3A+ monocytes was significantly reduced after plasmapheresis. These findings are consistent with the literature emphasizing the role of monocytes in the pathophysiology of AAV [11, 12]. In addition to activating neutrophils, ANCAs also activate monocytes. This may be related to the fact that monocytes can also express PR3 and MPO, albeit at a lower level than neutrophils. Notably, macrophages play an important role in the pathological process of AAV and are most enriched in glomeruli [13, 14]. This implies that plasmapheresis not only reduces active monocytes but also reduces macrophage infiltration through monocyte depletion.

Classical typing methods divided monocytes into three subpopulations based on the expression of CD14 and CD16. In healthy persons, ~80%–90% of monocytes are highly CD14 positive and CD16 negative (CD14++CD16−) classical monocytes. The remaining 10%–20% of monocytes are CD16 positive and can be subdivided into and intermediate (CD14++CD16+) and nonclassical (CD14+CD16++) monocytes, respectively [15]. In contrast to classical monocytes, CD16+ monocytes are perceived as proinflammatory due to their production of proinflammatory cytokines such as TNF-*α* and IL-1*β* [16]. Previous studies have also demonstrated a major role for CD16+ monocytes in AAV disease progression [12, 17].

The monocytes clusters we observed before and after plasmapheresis could be delineated into CD14+ monocytes, FCGR1A+ monocytes, and FCGR3A+ monocytes clusters. FCGR3A+ monocytes corresponded to CD16+ monocytes in the traditional classification. CD14+ monocytes and FCGR1A+ monocytes were the two clusters of CD14+ monocytes in the traditional classification according to the expression of FCGR1A. FCGR1A and FCGR3A encode CD64 and CD16, respectively. The proportion of FCGR1A+ monocytes and FCGR3A+ monocytes in monocytes and PBMCs decreased after plasmapheresis, while the proportion of CD14+ monocytes increased.

This provided a novel perspective to understand the cellular mechanisms underpinning the therapeutic effects of plasmapheresis. In our flow cytometry results, the positive correlation between CD16+ (FCGR3A+) monocytes and disease activity supports the notion that these cells contribute to the pathogenesis of AAV, as reported in other inflammatory diseases such as rheumatoid arthritis, coronary artery disease, and type 2 diabetes mellitus [18, 19]. Notably, CD16 is a transmembrane receptor whose ligand happens to be IgG, the major type of ANCAs. This implies that ANCAs may play a role in promoting inflammation by binding to CD16 on monocytes. FCGR3A+ monocyte reduction after plasmapheresis could thus signify a decrease in pro-inflammatory signaling and tissue damage, aligning with the enrichment analysis.

In contrast, the proportion of CD16-CD64+ (FCGR1A+) monocytes negatively correlated with disease activity may suggest FCGR1A+ monocytes reduce disease activity. CD64 is a transmembrane glycoprotein that forms a large immunoglobulin (Ig) superfamily with Fc*γ*RII (CD32) and Fc*γ*RIII (CD16) receptors, and it binds to IgG [20]. Studies have shown that CD64 plays a central role in macrophage immune complex clearance [21]. CD64 exerts its role by the internalization of immune complexes. In addition, it has been shown that overexpression of CD64 on monocytes and neutrophils in the blood of patients with sepsis, and ARDS is associated with reduced oxidative response [22]. In contrast, CD64 monocytes, although a small percentage of peripheral blood monocytes, represent a subset of monocytes that effectively interact with T cells in vitro and are the major source of interferon-*α* [23]. In our study, we found that the proportion of FCGR1A+ monocytes decreased after plasmapheresis in scRNA-seq. This may be related to the removal of interferon-*γ* and other inflammatory factors that can increase CD64 by plasmapheresis.

Taken together with our results, we conclude that monocytes play a critical role in the plasmapheresis treatment to AAV. In addition, we found a new method for monocyte clustering, which has the potential to be used as a marker for AAV activity.

The above implications of our findings may be far-reaching. The identification of monocyte subsets as potential biomarkers for disease activity and treatment response opens avenues for the development of AAV precise medicine approaches. Furthermore, these insights contribute to a broader understanding of the role of monocytes in autoimmune diseases, which has been a topic of considerable interest in recent immunological research [11, 24, 25].

However, our study still has some limitations. The sample size was modest, and the cross-sectional design limits our ability to draw definitive conclusions regarding the long-term effects of plasmapheresis on monocyte populations and disease activity. Longitudinal studies with larger cohorts are essential to validate our findings and explore the durability of the observed immunological changes. Moreover, functional assays are needed to determine the exact roles of the identified monocyte subsets in AAV pathogenesis and resolution, as well as their potential interactions with other immune cells, such as T cells and B cells, which have also been implicated in the disease process [3]. In addition, we did not perform scRNA-seq analysis in some AAV patients with similar disease activity who did not undergo plasmapheresis, and we have not yet performed cytokine levels, which would be areas of future work.

Based on our results, future studies should aim at exploring deeper molecular mechanisms and conducting cohort studies. Additionally, the exploration of how plasmapheresis influences other components of the immune system in AAV, such as the adaptive immune response could yield further insights into the comprehensive immunomodulatory effects of this treatment. The interplay between monocytes and lymphocytes, for instance, is critical in shaping the immune response, and alterations in monocyte subsets could have downstream effects on T-cell and B-cell function.

In conclusion, our study enhances the understanding of the immunological landscape in AAV and underscores the potential of new monocyte profiles as a biomarker for assessing disease activity and response to plasmapheresis. The insights gained from our research not only have immediate clinical relevance but also provide a foundation for future studies.

## Data Availability

For raw data or additional Information, please contact the corresponding author.

## Supporting Information

Supporting Information 1 Figure S1: Quality control of scRNA-seq. (A) pretreatment scRNA-seq data before quality control; (B) post-treatment scRNA-seq data before quality control; (C) pretreatment scRNA-seq data after quality control; (D) post-treatment scRNA-seq data after quality control. Figure S2: Cluster dendrogram of PBMCs. Figure S3: Proportion of cell clusters per sample. (A) The proportion of the PBMCs subpopulation in each sample; (B) The proportion of monocyte subsets in each sample. Figure S4: Enrichment analysis of up-regulated DEGs before and after treatment in monocytes clusters. Figure S5: Enrichment analysis of down-regulated DEGs before and after treatment in monocytes clusters. Figure S6: Cell annotation of PBMCs under the new monocyte classification.

Supporting Information 2 Table S1: Birmingham systemic vasculitis activity score (BVAS).

Supporting Information 3 Table S2: Demographic and clinical information.
