# Supplementary material for: Immune Landscape Variation in Antineutrophil Cytoplasmic Antibody-Associated Vasculitis Circulation Before and After Plasmapheresis by Single-Cell Transcriptome
Source: Mediators Inflamm. 2025 Apr 10;2025:5531382. doi: 10.1155/mi/5531382 (PMC12006691; doi:10.1155/mi/5531382)
Supplement: Supporting Information 1 — Figure S1: Quality control of scRNA-seq. (A) pretreatment scRNA-seq data before quality control; (B) post-treatment scRNA-seq data before quality control; (C) pretreatment scRNA-seq data after quality control; (D) post-treatment scRNA-seq data after quality control. Figure S2: Cluster dendrogram of PBMCs. Figure S3: Proportion of cell clusters per sample. (A) The proportion of the PBMCs subpopulation in each sample; (B) The proportion of monocyte subsets in each sample. Figure S4: Enrichment analysis of up-regulated DEGs before and after treatment in monocytes clusters. Figure S5: Enrichment analysis of down-regulated DEGs before and after treatment in monocytes clusters. Figure S6: Cell annotation of PBMCs under the new monocyte classification. [file 5531382.f1.docx]

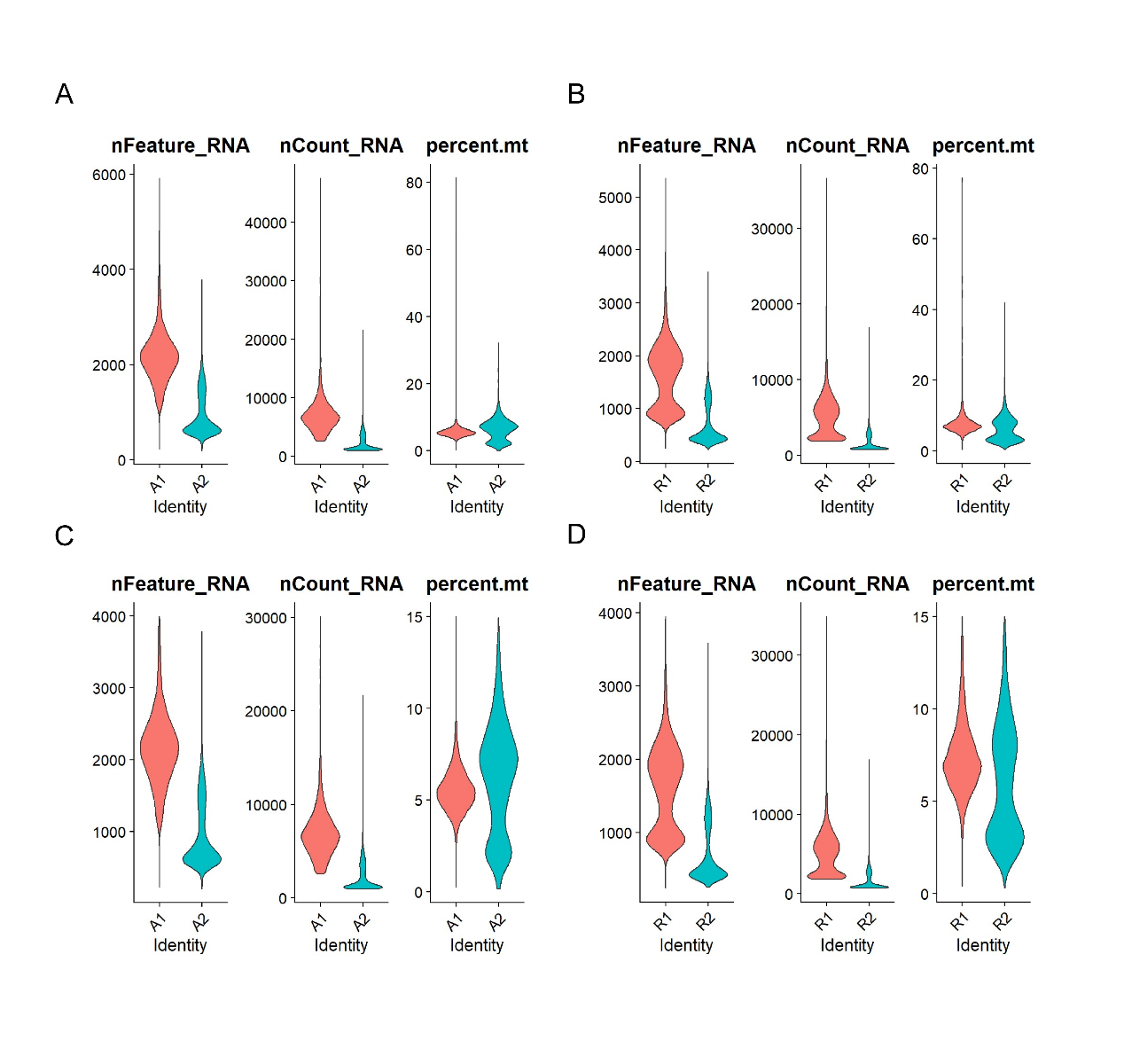


**Supplementary Figure 1. Quality Control of scRNA-seq.**

(A) pretreatment scRNA-seq data before quality control; (B) post-treatment scRNA-seq data before quality control; (C) pretreatment scRNA-seq data after quality control; (D) post-treatment scRNA-seq data after quality control.


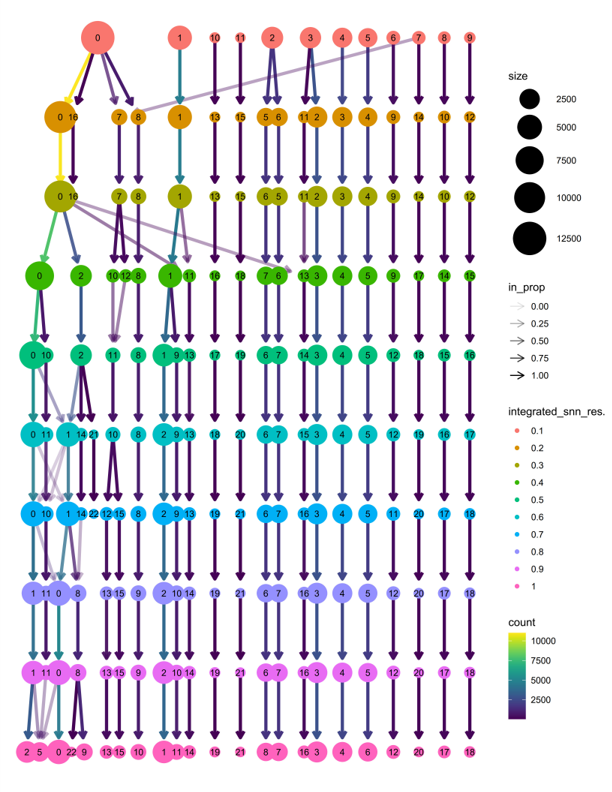


**Supplementary Figure 2. Cluster dendrogram of PBMCs.**

**
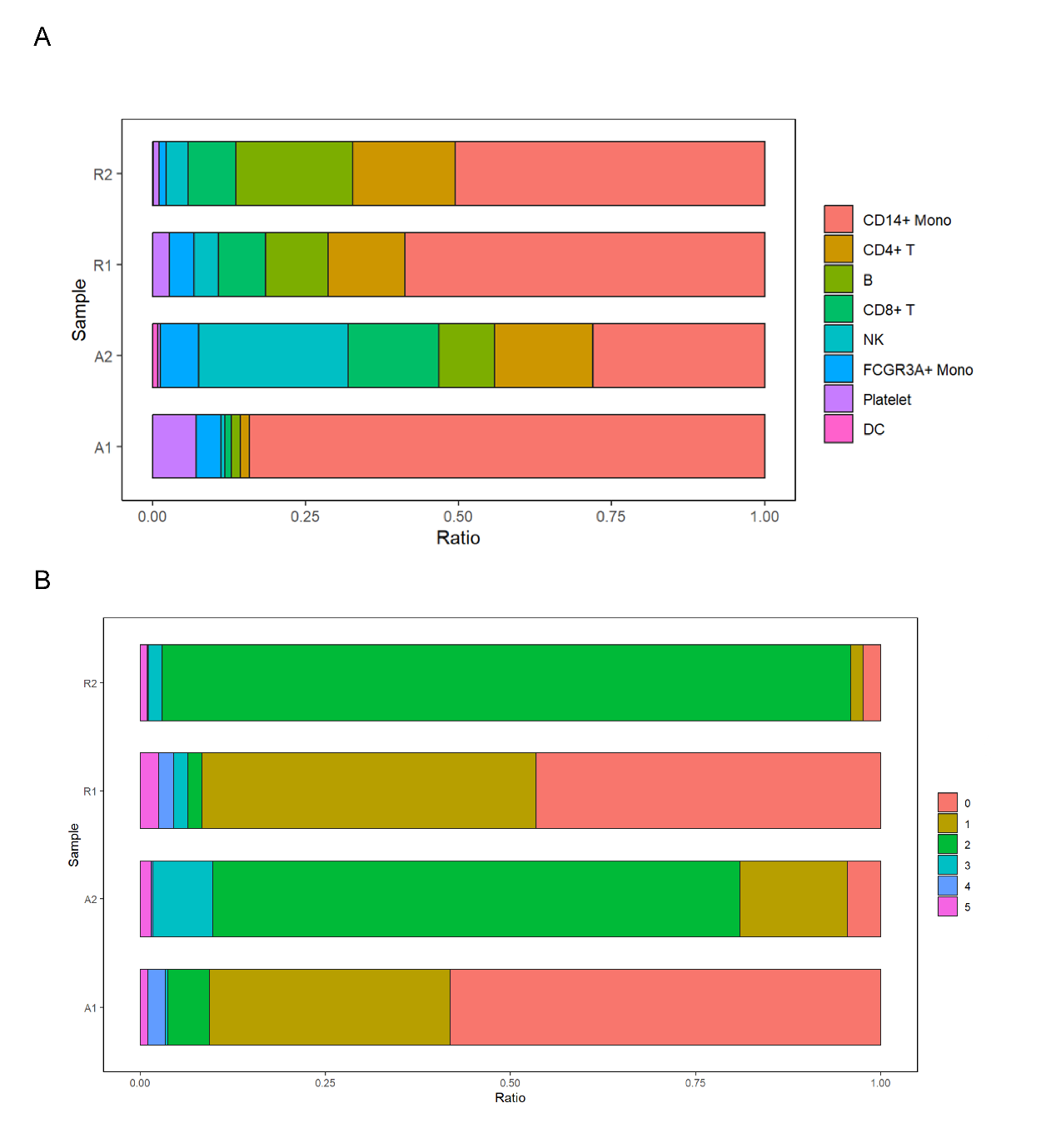
**

**Supplementary Figure 3. Proportion of cell clusters per sample.**

(A) The proportion of the PBMCs subpopulation in each sample; (B) The proportion of monocyte subsets in each sample.


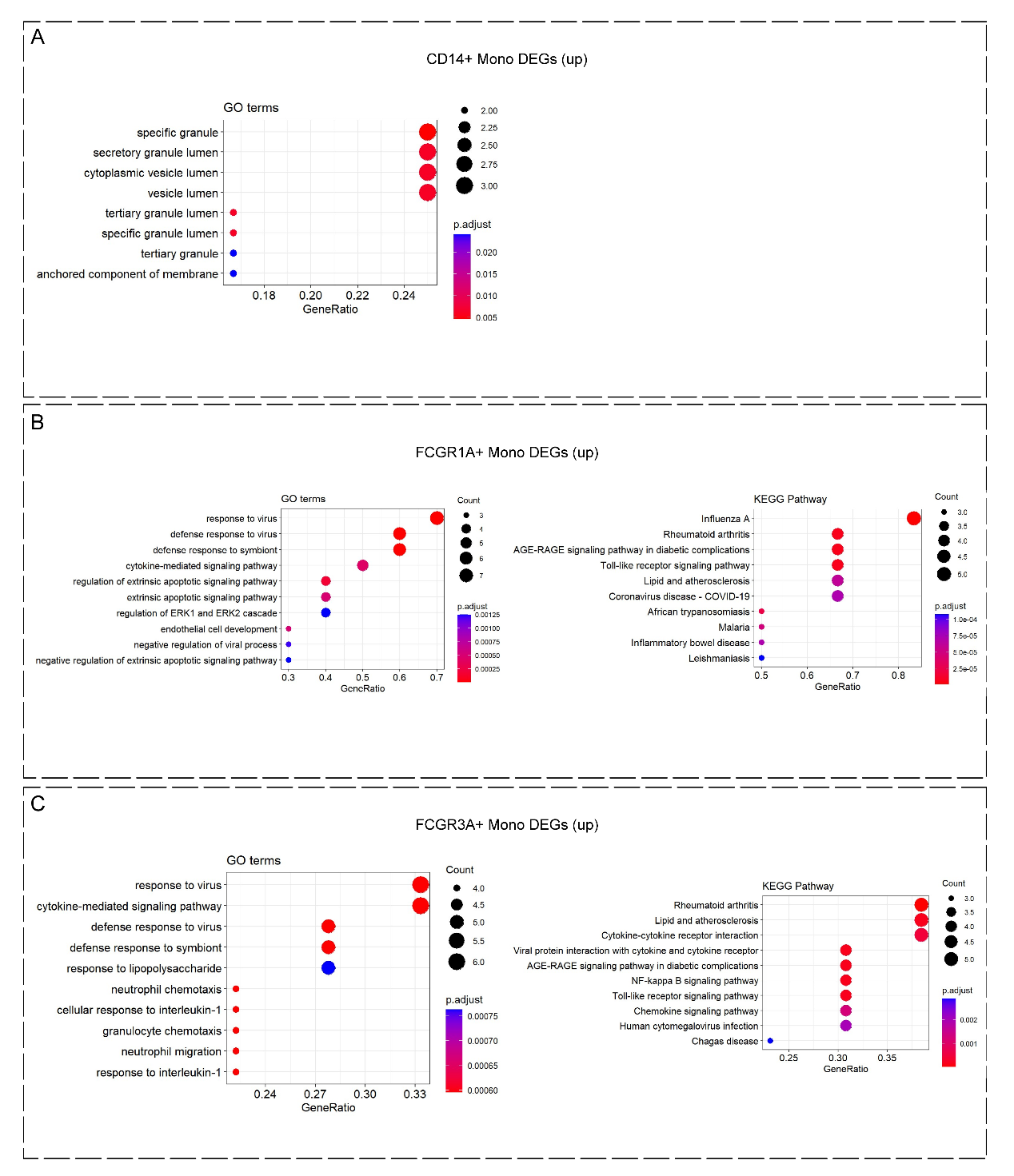


**Supplementary Figure 4.** **Enrichment analysis of up-regulated DEGs before and after treatment in monocytes clusters.**


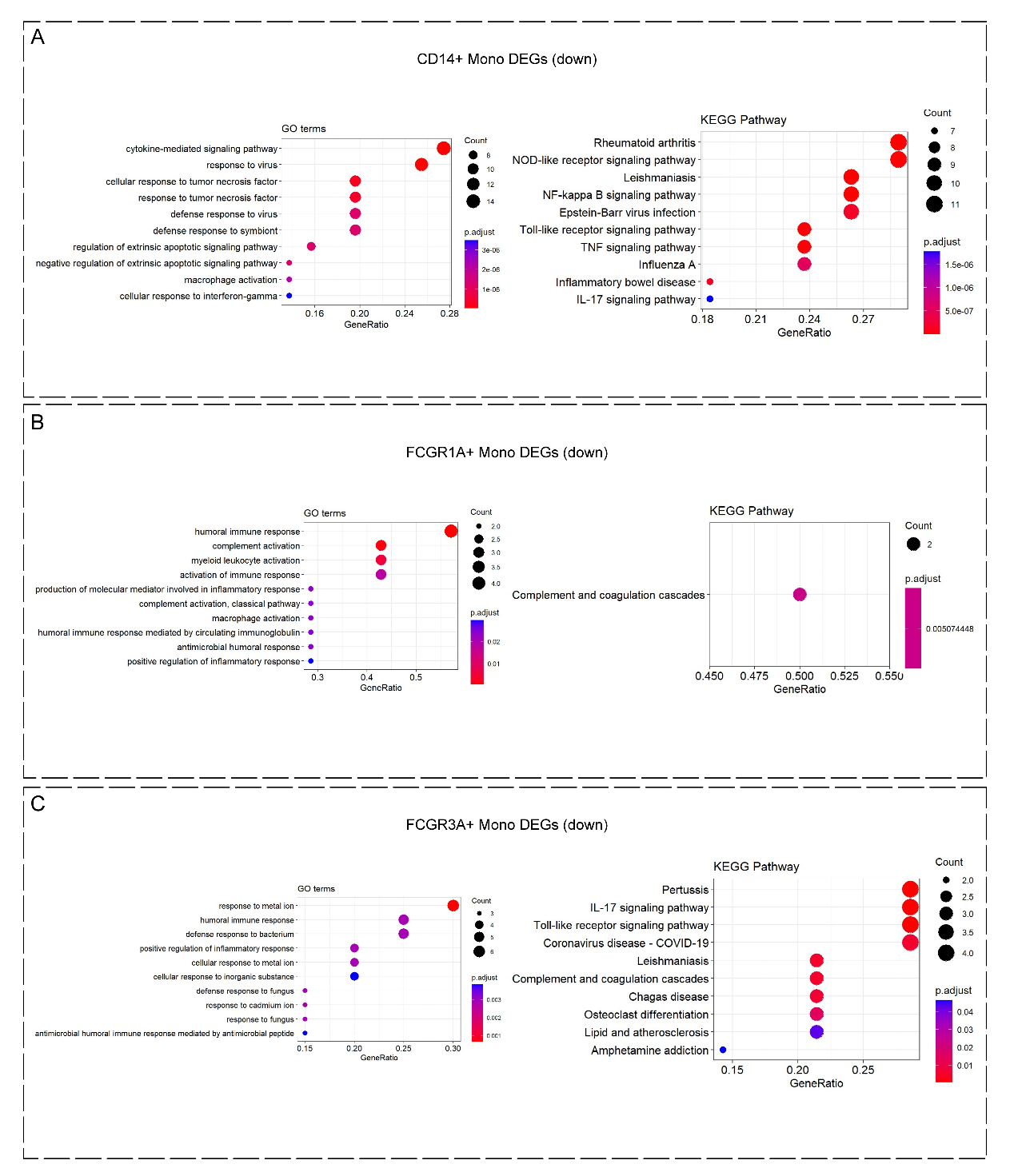


**Supplementary Figure 5.** **Enrichment analysis of down-regulated DEGs before and after treatment in monocytes clusters.**


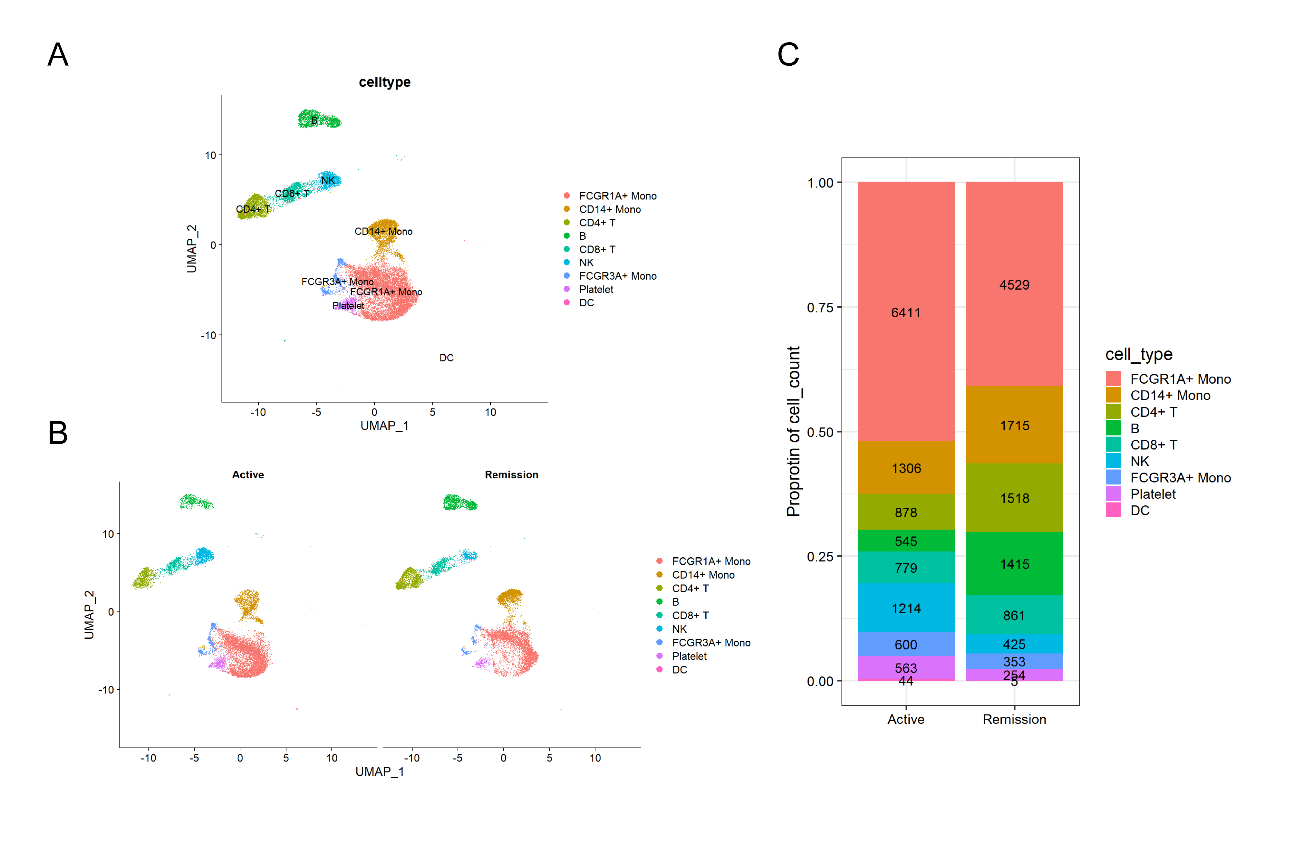


**Supplementary Figure 6. Cell annotation of PBMCs under the new monocyte classification.**
